# Effects of preoperative chronic hypoxemia on geriatrics outcomes after hip arthroplasty: A hospital-based retrospective analysis study

**DOI:** 10.1097/MD.0000000000006587

**Published:** 2017-04-14

**Authors:** Furong Zhang, Ruqiang Zhang, Liang He, Jianwei Yin, Fang Wang, Junmin Li

**Affiliations:** a Department of Anesthesiology; b Department of Orthopedics, Yan’an Hospital of Kunming City, Kunming Medical University, Kunming, China.

**Keywords:** chronic hypoxemia, high altitude, hypoxic preconditioning, perioperative medicine, postoperative complications

## Abstract

The partial pressure of oxygen decreases as altitude increases, the preoperative chronic hypoxemia (CH) may have a plausible clinical impact. Risk factors for postoperative serious adverse events (pSAEs) in patients living in high altitudes during primary hip arthroplasty (HA) are not clear.

This is an observational study embracing patients from January 1, 2011 to December 31, 2015 at Yan’an Hospital of Kunming City, a 1338-bed municipal teaching hospital of Kunming Medical University. Univariate analysis revealed that significant differences between patients with and without preoperative CH occurred in intraoperative hypotension (77 [33%] vs 34 [47%], *P* = .040) and that significant differences between patients with and without pSAEs occurred in following variables: preoperative CH (32 [57%] vs 199 [80%], *P* < .001), intraoperative hypotension (37 [66%] vs 74 [30%], *P* < .001), highest noradrenaline support (.09 [.01–.21] vs .03 [.01–.05] μg/kg/min, *P* < .001), higher application of general anesthesia (15 [27%] vs 29 [12%], *P* = .004), and lower of combined-spinal epidural anesthesia (CSEA) (21 [37%] vs 165 [66%], *P* < .001). The general anesthesia and intraoperative hypotension remained the independent risk factors for pSAEs (*P* < .05), while the preoperative CH presented by decreasing its risk (*P* < .05).

This study suggests that various intraoperative events including general anesthesia, hypotension were risk factors for the development of pSAEs. Preoperative CH, presenting with decreased incidence of intensive care unit (ICU) admission and pSAEs, may mimic hypoxic preconditioning in organic protection, for which further study is needed to uncover the underlying mechanisms.

## 1 Introduction

Hypobaric hypoxia occurs naturally at high altitudes, and total atmospheric pressure decreases as altitude increases, causing a lower partial pressure of oxygen.^[1–3]^ The higher incidence of chronic hypoxemia (CH) geriatric patients in high altitude is largely known, may have a plausible clinical impact.^[3]^ As it known, patient- and surgery-related factors have been associated with a plausible decrease in the quality of life during hospital and even later.^[4,5]^ A successful hip arthroplasty (HA) can modify the quality of postoperative life and relieve pain for patients who suffer severe hip osteoarthritis or fracture.^[6,7]^ HAs are performed in a wide variety of patients, ranging from those requesting surgery to facilitate their highly active lifestyle to those who require surgery in order to perform the routine activities of daily living.^[4]^ Age, types of anesthesia, hypoxemia, anemia, and intraoperative hypotension appear to develop the postoperative outcomes in seniles after orthopedic surgery.^[8,9]^ Surprizingly, few studies have tested the associations between anesthesia, preoperative CH, high altitude, age, perioperative treatment, and postoperative serious adverse events (pSAEs) during HA. Herein, with the increasing international recognition that perioperative management approach does affect patients’ outcomes,^[4,10]^ we conducted a retrospective analyses of elderly patients suffering primary HA in altitude area of Kunming, China.

The preoperative CH, adaptive response to evolution of high altitude, may mimic the hypoxic preconditioning, which is confirmed as organic protection including brain and heart from laboratory studies to some clinical trials.^[11–15]^ We hypothesized that preoperative CH, adaptive response to high altitude, is significantly associated with the development of pSAEs in primary HA for elderly native patients in high altitude areas. The audit was performed as the following aims: provide a detailed description of all patients consecutively admitted to this teaching hospital in the period time of HA and discharge; give the enhanced importance of preoperative and intraoperative characteristics; and explore the association between CH and the occurrence of pSAEs.

## 2 Methods

### 2.1 Patients and study design

The local Institutional Review Board for Clinical Investigations at Yan’an Hospital of Kunming City approved the retrospective cohort study. Inclusion criteria were elderly patients without limitation of American Society of Anesthesiologists physical status or gender, living Kunming (the altitude is about 1850 m) over 10 years, aged above 69 years undergoing elective primary HA under anesthesia. Exclusion criteria were: cyonosis; acute stroke; preoperative dementia; revision, resurfacing, or bilateral procedures; and intraoperative blood loss or transfusion above 600 mL, without necessary details in recordings.

We retrospectively searched this hospital paper documents for all HA coded between January 2011 and December 2015.

We collected arterial partial of oxygen (PaO_2_) or saturation of pulse oxygenation (SpO_2_) of patients, gender, age, preoperative hemoglobin (Hb), American Society of Anesthesiologists physical status, and comorbidity. Intraoperative parameters were type of joint (cemented or not), anesthesia method of general, epidural or combined-spinal epidural anesthesia (CSEA), duration of HA, mean arterial pressure (MAP), vasopressor, blood loss, and transfusion of red blood cell. Arterial blood gas was obtained upon admission, 30 minutes before anesthesia and after incision analyzed, if needed. The postoperative variables were intensive care unit (ICU) admission, SAEs including pulmonary embolism, malignant arrhythmia needing cardiologist consultation and delirium, pLOS, and mortality before discharge. Given the clinical determination of hypoxemia for patients with lung injuries varies, but typical values are PaO_2_ < 60 mm Hg and arterial oxygenation (pulse oximetry) <88%.^[16]^ But in this study, the preoperative CH was defined as the SpO_2_ was less than 88% or PaO_2_ from arterial blood gas was less than 60 mm Hg, which was adjusted by altitudes.

### 2.2 Statistical analysis

This is the first observational study aiming to investigate the effect of preoperative CH on pSAEs in natives undergoing HA in high altitude area. The data were firstly analyzed in terms of cohort study following patients with or without preoperative CH, then the same data were analyzed in terms of case–control study for the patients with or without pSAEs. In view of limitation sample size in patients included, all the episodes over 5-year period were analyzed. The multivariate analysis with preoperative and intraoperative variables as potential risk factors for pSAEs and ICU admission was performed by logistic regression. In addition, binary outcomes were analyzed by Chi-square tests. According to the Kolmogorov–Smirnov test, a Gaussian distribution of continuous data were tested to be conducted by *t* tests, or the Mann–Whitney *U* tests; SPSS for Windows (Version 16.0, SPSS, Inc., Chicago, IL) was used to perform all the analysis. A *P* value less than .05 was considered to be significantly difference.

## 3 Results

**Figure 1:**
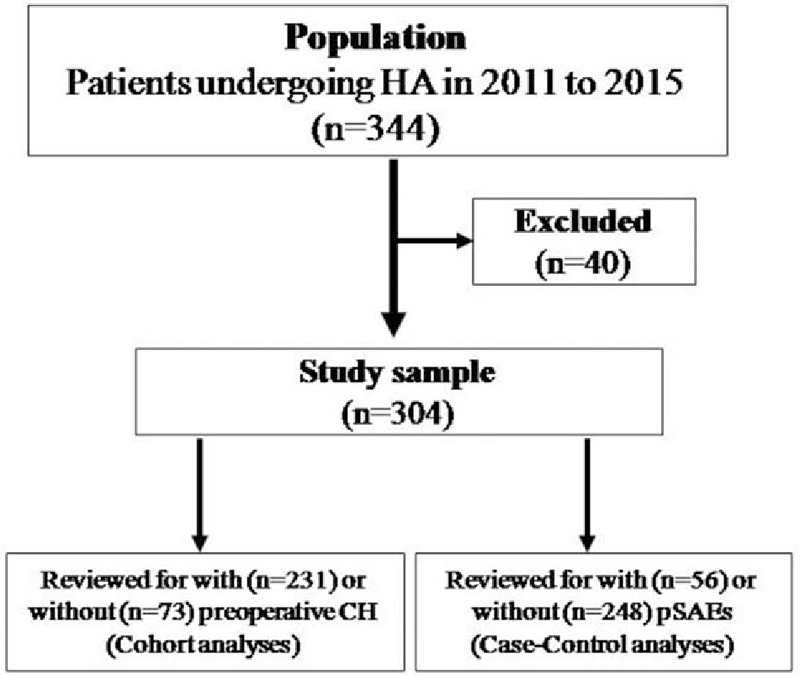
Study flowchart.

From January 2011 to December 2015, a search of records 344 patients for primary HA at Yan’an Hospital of Kunming City, 40 were excluded (Fig. 1). Of the 304 patients included in this study, 56 (18%) developed SAEs before discharge. The presentation of pSAEs were malignant arrhythmia, delirium, pulmonary embolism, and death in 23 (41%), 20 (36%), 8 (14%), and 5 (9%), respectively. Patients with preoperative CH received similar percentage in type of anesthesia (*P* > .05) and vasopressor support (*P* = .813), lower incidences of intraoperative hypotension (77 [33%] vs 34 [47%], *P* = .040), lower levels of SpO_2_ (82 ± 5 vs 92 ± 4, *P* < .001) and PaO_2_ (48 ± 5 vs 62 ± 5, *P* < .001), and less noradrenaline support (.03 [.01–.06] vs .17 [.01–.30] μg/kg/min, *P* < .001). Perioperative characteristics in patients with or without preoperative CH are given details in Table 1.

**Table 1:**
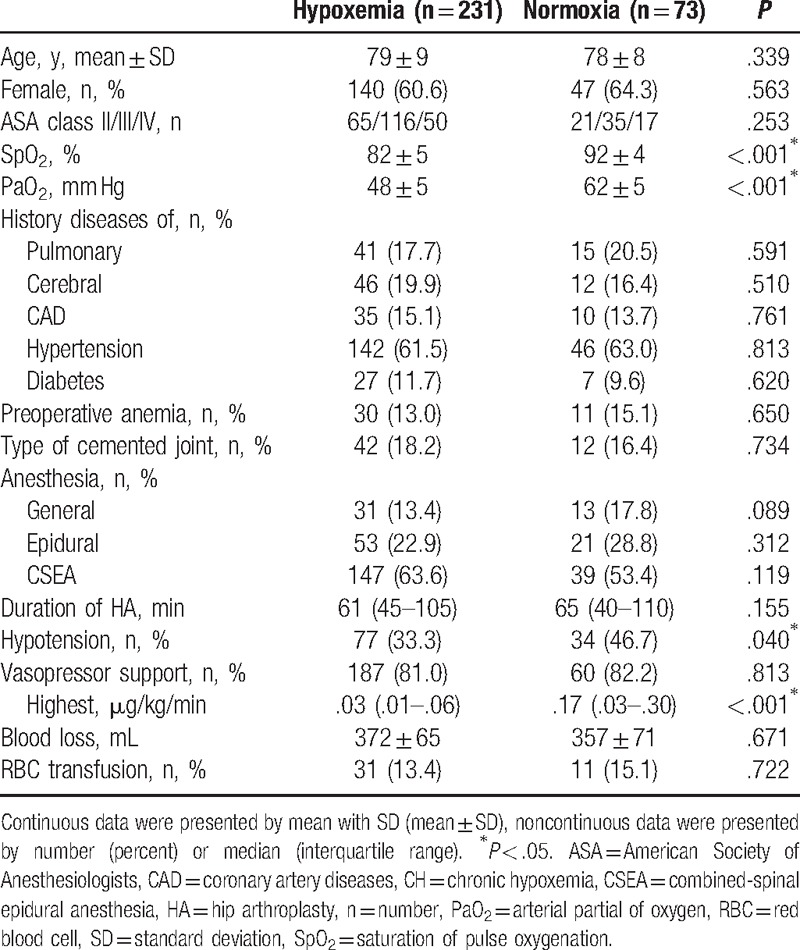
Perioperative characteristics in patients with or without preoperative CH.

Characteristics of patients with or without pSAEs are given details in Table 2. Patients with pSAEs received: lower ratios of preoperative CH (32 [57.1%] vs 199 [80.2%], *P* < .001); higher application of general (26.8% vs 11.7%, *P* = .004) and epidural anesthesia (35.7% vs 21.8%, *P* = .028), but less utilization of CSEA (37.5% vs 66.5%, *P* < .001); similar ratios of vasopressor support (*P* = .570), higher incidences of intraoperative hypotension (37 [66.1%] vs 74 [29.8%], *P* = .001), but highest noradrenaline support (.09 [.01–.21] vs .03 [.01–.05] μg/kg/min, *P* < .001); and lower levels of SpO_2_ and PaO_2_ (*P* < .01).

**Table 2:**
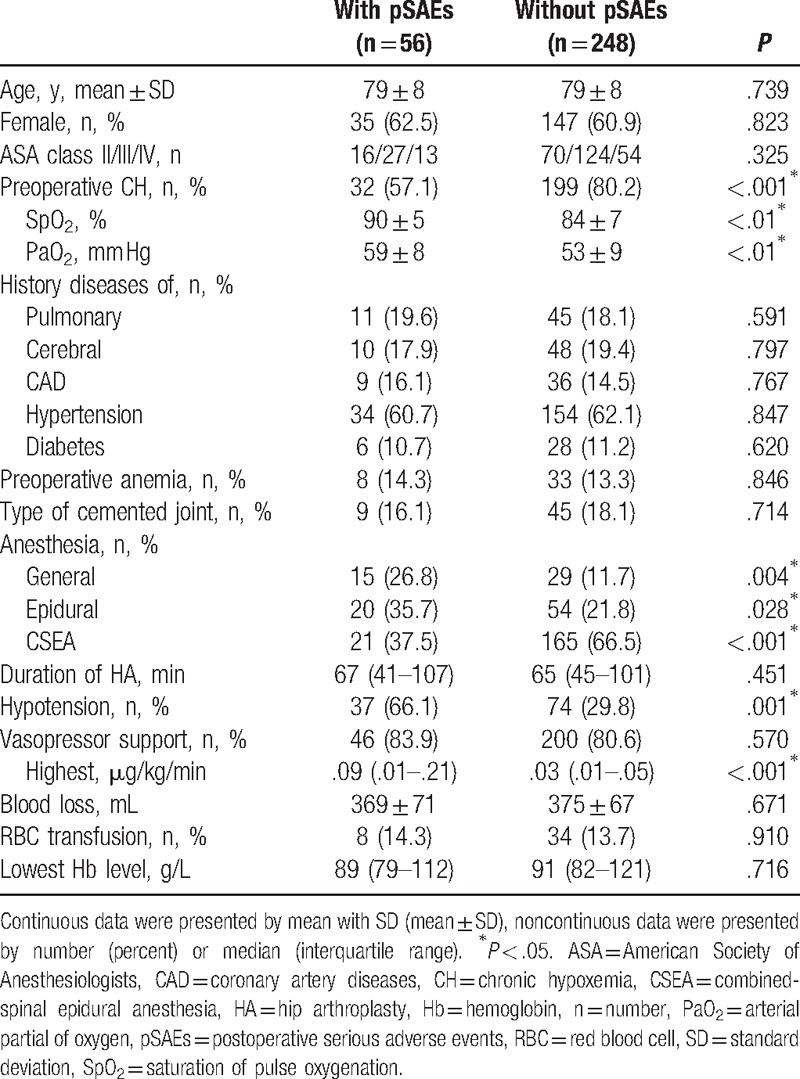
Characteristics of patients with or without pSAEs.

The association of preoperative CH and outcomes following HA are provided in Table 3. As it shown in this table, preoperative CH decreased pSAEs by 85% with odds ratio (OR) of .15% and 95% confidence interval (CI) from .08 to .28 (*P* < .001). Patients with CH were presented with less pLOS (10 vs 15, *P* < .05).

**Table 3:**
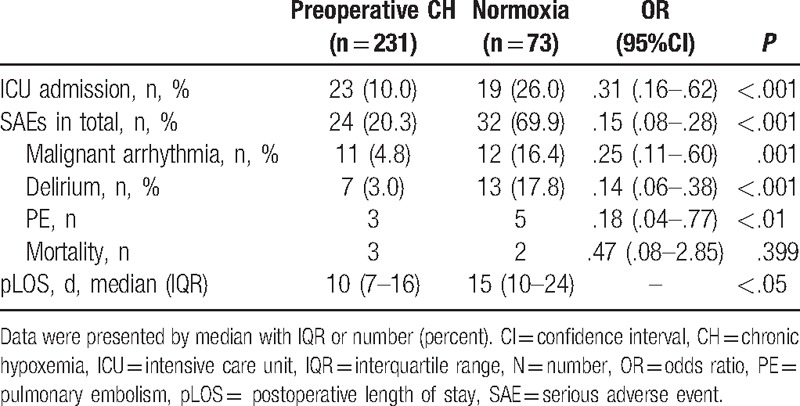
Association of preoperative CH and outcomes after HA.

The multivariate analysis with preoperative and intraoperative variables as potential risk factors for pSAEs was performed by logistic regression. The general anesthesia and intraoperative hypotension remained the independent risk factors for pSAEs with OR of 4.08 (95%CI: 2.12–8.91) and 4.92 (95%CI: 1.91–11.29), respectively, while the preoperative CH presented by decreasing its risk with OR = .57 (95%CI: .21–.93) (*P* < .05, data were similar with those reported as before in our study^[17]^).

## 4 Discussion

In this retrospective analysis of 304 consecutive elderly patients undergoing HA, about 14% of the geriatrics were transferred to ICU and 18% developed pSAEs before discharge. Although perioperative characteristics excluding hypotension were similar between groups, elderly patients with and without CH differed significantly in pSAEs including ICU admission, malignant arrhythmia, delirium, and pulmonary embolism, as well as the pLOS. With regard to intraoperative variables, general anesthesia, low MAP lasting over 3 minutes, and high vasopressor requirements were significant intraoperative risk factors. Although in the multivariate analysis, both general anesthesia and intraoperative hypotension remained independent, suggesting that general anesthesia technique and intraoperative hypotension lasting over 3 minutes are the independent risk factors for the development of postoperative complications (ICU admission and pSAEs) before discharge in elderly patients in high altitude.

The intraoperative hypotension increased the incidence of pSAEs by more than 4-fold in this observational study. There is dispute in relationship between intraoperative hypotension and adverse outcomes after noncardiac surgery,^[18,19]^ as it was not reported in geriatrics in high altitude. To some extent, the MAP values decreasing more than 30% from baseline was associated with a higher risk of postoperative ischemic stroke.^[20]^ And when MAP was less than 60 mm Hg, patients have been reported as suffering from acute kidney injury after noncardiac surgery.^[21]^ Hallqvist et al found a reduction in systolic blood pressure (SBP) of more than 50% from baseline lasting more than 5 minutes increased the incidence of myocardial damage.^[22]^ Therefore, stable intraoperative blood pressure without fluctuations is likely to be beneficial to the patient,^[23]^ as it was confirmed in the present retrospective observational study.

Intriguingly, preoperative CH was presented by decreasing pSAEs. There is evidence of natural selection centered around the hypoxia-inducible factor pathway in high-altitude-adapted natives, but the underlying mechanisms, including the possible role of hypoxia-signaling pathways, remain to be resolved.^[24]^ When it comes to anesthetic techniques, we have detected CSEA is far superior to general anesthesia in geriatric patients with CH undergoing HA.^[17,25]^

Conclusion of our previous study was similar to that of a retrospective analysis with a large sample size published in the Lancet.^[26]^ Consequently, application of CSEA is popular in our hospital.

This retrospective observational study has some weaknesses. First, the data collected are observational. Second, with regard to preoperative CH may mimic hypoxic preconditioning and decrease pSAEs, of which mechanisms were not demonstrated. Third, compared with a randomized controlled clinical trial, it is possible that the results suffered some bias.

We conclude that intraoperative hypotension and general anesthesia are independent predictors of a poor outcome in geriatric patients with preoperative CH undergoing primary HA in routine practice in high altitude area of China and that CSEA with stable hemodynamics is feasible in this setting. We must now postulate that more high-quality prospective studies are required to confirm these findings, establish evidence-based clinical guidelines, and unravel the underlying mechanisms.
